# Community-Acquired Methicillin-Resistant *Staphylococcus aureus,* Finland

**DOI:** 10.3201/eid0806.010313

**Published:** 2002-06

**Authors:** Saara Salmenlinna, Outi Lyytikäinen, Jaana Vuopio-Varkila

**Affiliations:** * National Public Health Institute, Helsinki, Finland

**Keywords:** Community-acquired, MRSA

## Abstract

Methicillin-resistant *Staphylococcus aureus* (MRSA) is no longer only hospital acquired. MRSA is defined as community acquired if the MRSA-positive specimen was obtained outside hospital settings or within 2 days of hospital admission, and if it was from a person who had not been hospitalized within 2 years before the date of MRSA isolation. To estimate the proportion of community-acquired MRSA, we analyzed previous hospitalizations for all MRSA-positive persons in Finland from1997 to 1999 by using data from the National Hospital Discharge Register. Of 526 MRSA-positive persons, 21% had community-acquired MRSA. Three MRSA strains identified by phage typing, pulsed-field gel electrophoresis, and ribotyping were associated with community acquisition. None of the strains were multiresistant, and all showed an *mec* hypervariable region hybridization pattern A (HVR type A). None of the epidemic multiresistant hospital strains were prevalent in nonhospitalized persons. Our population-based data suggest that community-acquired MRSA may also arise de novo, through horizontal acquisition of the *mecA* gene.

Methicillin-resistant *Staphylococcus aureus* (MRSA) is an important cause of nosocomial infections worldwide. Recent studies suggest that the epidemiology of MRSA may be changing, as the isolation of MRSA is no longer limited to hospitalized patients or persons with predisposing risk factors (*1*–*4*). However, the prevalence of MRSA colonization in healthy persons in the community has been shown to be low, even when MRSA is highly endemic in hospital settings (*5*).

Nosocomial MRSA strains in the community, including nursing homes and other nonacute-care facilities, may be transmitted by discharged patients and health-care workers (*6*,*7*). Whether strains of MRSA in the community also arise de novo, as a consequence of horizontal acquisition of the *mecA* gene, is unclear. The transfer of *mecA* DNA to a susceptible *S. aureus* strain has occurred in vitro (*8*) and recently in a hospitalized patient during antibiotic treatment (*9*). The mechanism of transfer of *mec* DNA from a donor to a recipient is not completely understood. However, the excision and integration of the *mec* DNA from and to the chromosome are apparently catalyzed by cassette chromosome recombinases A and B (Ccr A and B) coded by *mec*-associated genes (*ccrA* and *B*), with homology to the invertase-resolvase family of DNA recombinases (*10*).

In Finland, the prevalence of MRSA has remained low, although several hospital epidemics have occurred in the last decade (*11*). We recently recognized two distinct groups of MRSA, one representing multiresistant epidemic strains and the other only β-lactam-resistant strains. These two groups also showed differences in ribotypes and *mec* determinant profiles (*12*). The aim of this study was to estimate the proportion of community-acquired MRSA by analyzing the hospital contacts of persons from whom MRSA was found from 1997 to 1999. We also compared the MRSA isolates in persons with and without hospital contact in terms of strain type (determined by phage typing, pulsed-field gel electrophoresis [PFGE], and ribotyping), antibiotic resistance, and *mec* determinant profile.

## Materials and Methods

### Surveillance and Typing Scheme of MRSA

Finnish microbiology laboratories report (generally electronically) all MRSA isolates to the National Infectious Disease Register at the National Public Health Institute (KTL). The KTL records the date, source of specimen, and the patient’s birth date, sex, and place of treatment. Using this information and a time interval of 36 months, multiple isolations from the same person are deleted from the database. The microbiology laboratories also send the MRSA isolates to the Laboratory of Hospital Bacteriology at KTL for further analysis. Phage typing, PFGE, and antimicrobial drug susceptibility testing were performed as described (*11*). In brief, phage typing was performed with the universal set of phages (*13*) at 1x and 100x routine test dilutions, both with and without heat treatment of bacteria (*14*). Antimicrobial drug susceptibilities were tested by the disk diffusion method according to guidelines recommended by the National Committee for Clinical Laboratory Standards (NCCLS). The antibiotics tested were oxacillin, ampicillin, penicillin, cephalexin, cefuroxime, gentamicin, tobramycin, erythromycin, clindamycin, chloramphenicol, ciprofloxacin, rifampicin, fusidic acid, mupirocin, and vancomycin. MICs of oxacillin were determined by E-test (AB Biodisk, Solna, Sweden) according to manufacturer’s instructions. If the oxacillin MIC was <64 μg/mL, methicillin resistance was verified with the MRSA screen test (Denka Seiken, Japan) or *mecA*-polymerase chain reaction (*15*). For PFGE, genomic DNA prepared in agarose blocks was digested with *Sma*I restriction endonuclease, and chromosomal fragments were separated with a Chef DR III (Bio-Rad Laboratories, Hercules, CA) for 24 hours with initial and final switching times of 10 seconds and 60 seconds, respectively. PFGE profiles differing by fewer than four bands were interpreted as identical or closely related (*16*). Bionumerics software, with Dice coefficient and unweighted pair group matching average for clustering, and optimization and tolerance values of 1% and 1–2%, respectively, were used to verify the relatedness of PFGE profiles. MRSA isolates sharing identical or closely related PFGE profiles and phage types were considered to be the same strain type. If the PFGE profiles were related, but the phage types were different, ribotyping with one to three restriction enzymes (*Hin*dIII*, Eco*RI*, Cla*I*)* was performed to verify the relatedness of isolates (*11*). Riboprofiles with fewer than four bands’ difference were regarded as identical or closely related. The genomic variation within the *mec* determinant hypervariable region (HVR) was analyzed for a subset of MRSA isolates as described (*12*). In brief, genomic DNA digested with *Eco*RI and *Bgl*II restriction endonucleases was hybridized with two probes prepared from plasmid pBBB30 (*17*) and was recognized as the *mec* hypervariable region.

### Study Population, MRSA Isolates, and Definitions

All MRSA-positive persons in Finland from 1997 to 1999 and their MRSA isolates (one from each) were included in the study. A sporadic strain was defined as a strain type isolated from one person only. An MRSA isolate was defined as hospital acquired if the MRSA-positive specimen was obtained 2 days after hospital admission, and for discharged patients, if the patient had been hospitalized within 2 years before the date of MRSA isolation.

### Previous Contacts with a Health-Care Facility

National identity codes for each person with an MRSA-positive culture were obtained either through the primary diagnostic laboratory or from the infection control nurse of the health-care facility. Based on the first isolation date of MRSA and the national identity code, data on previous hospitalizations within 2 years before the MRSA isolation date were retrieved from the National Hospital Discharge Register (HILMO). The HILMO is a civil register comprising comprehensive health-care records, provided by all hospitals and health-care centers in Finland, including outpatient surgery. Each report to the register includes patient identity information, admission and discharge dates, a code of the health-care provider, type of service, specialty, the place (home or institution) from which the patient came to the institution, and data on surgical procedures.

Additional background information was collected for persons for whom no HILMO reports could be found by sending questionnaires to infection-control nurses at relevant health-care facilities. The information collected included: 1) whether the MRSA-positive person was a patient or a staff member, 2) whether the specimen was taken on clinical or screening basis, and 3) whether the screening sample was taken because of a hospital contact abroad or because of an epidemic situation.

### Statistical Analysis

For categorical variables, proportions were compared by the chi-square test with Yates correction or Fisher’s exact test, as appropriate. The means and medians of the continuous variables were compared by the Student’s *t* test or Mann-Whitney *U* test, depending on the sample distribution.

With approval from the Ministry of Social Affairs and Health and the Finnish data protection authority, the National Research and Development Centre for Welfare and Health gave permission to use the data from the HILMO register.

## Results

From 1997 to 1999, 520 MRSA isolates were sent to the National Infectious Disease Register; the annual incidence ranged from 2.3 to 4.1/100,000 persons. The Laboratory of Hospital Bacteriology received MRSA isolates from 529 persons. Three persons did not have a Finnish national identity code and were excluded from the study. The median age of the 526 persons was 51 years (range 0–96), and 291 (55%) were male.

### Contacts with Health-Care Facilities

Of the 526 MRSA-positive persons, 108 (21%) did not have any verified link to health-care facilities 2 years before the MRSA isolation date, including 17 persons whose MRSA was isolated within 2 days of hospital admission. Their MRSA isolates were classified as community acquired. Specimens were taken from 69 persons; 37 persons were specifically screened for MRSA. Of those 37, 21 had a known MRSA contact in their family, 13 were otherwise exposed to a known MRSA carrier, and 3 were known to have carried MRSA previously.

The HILMO register and the questionnaire survey showed 418 (79%) persons who had at least one connection to a hospital, and their MRSA isolates were classified as hospital acquired. According to the HILMO register, 376 persons were hospitalized in Finnish hospitals within 2 years before the MRSA isolation date: 156 hospitalized patients with MRSA isolated 2 days after hospital admission and 220 discharged patients. The time frame between the MRSA isolation date and the previous hospitalization for the discharged patients was <6 months for 156 (71%) patients, 6 to 12 months for 36 (16%), and >12 months for 28 (13%) patients. The questionnaire survey identified 42 additional persons who had an obvious contact with a hospital, including 23 staff members (12 working in a Finnish health-care facility and 11 who had recently worked abroad) and 19 patients, 15 of whom had recently been hospitalized outside Finland.

The median age of the persons who did not have a contact with a health-care facility was lower than that of persons who had contact (34 vs. 58 years, p<0.01). The proportion of children <15 years of age was higher in community-acquired than in hospital-acquired MRSA strains (27 [25%] of 108 vs. 31 [7%] of 418, p<0.01).

### Strain Types

Among the 526 MRSA isolates, our typing scheme showed 84 strain types, 56 (67%) of which were sporadic and 28 (33%) shared by at least two persons. The distribution of sporadic (in total 56 [11%] of 526) and shared (in total 470 [89%] of 526) strain types was similar in persons with and without connections to health-care facilities (Table 1).

**Table 1:** Distribution of sporadic strains of methicillin-resistant *Staphylococcus aureus* (MRSA) and strains shared by at least two persons and contact with a health-care facility, Finland

	Persons with hospital contact (%)	Persons without hospital contact (%)
	n=418	n=108
Sporadic strain types	43 (10)	13 (12)
Strain types shared by at least two persons	375 (90)	95 (88)

Fourteen strain types, each of which were isolated from >10 persons, represented 421 (80%) of 526 MRSA isolations (Table 2, Figure). Three of the 14 most common strain types were more likely to be found in persons who did not have a contact with a health-care facility than in those who had such a contact: Mikkeli clone (41 [38%] of 108 vs. 75 [18%] of 418, p<0.01), E31 (10 [9%] of 108 vs. 6 [1%] of 418, p<0.01), and E22 (8 [7%] of 108 vs. 7 [2%] of 418, p<0.01).

**Table 2:** Methicillin-resistant *Staphylococcus aureus* (MRSA) strains found in >10 persons in relation to contact with a health-care facility, 1997–1999, Finland^a^

Strain type	Persons with hospital contact (%)	Persons without hospital contact (%)	Total	Multiresistance^b^	HVR type	Yr first identified in Finland
Mikkeli clone (O11, E12)	75 (65)	41 (35)	116	No	A	1993
E1	50 (98)	1 (2)	51	Yes	D	1992
E24	49 (98)	1 (2)	50	Yes	C	1998
E5 (UK EMRSA 16)	36 (100)	0 (0)	36	Yes	C	1995
Kemi clone	25 (71)	10 (29)	35	No	A	1996
E27	17 (65)	9 (35)	26	No	A	1997
E31	6 (38)	10 (63)	16	No	A	1997
E22	7 (47)	8 (53)	15	No	A	1997
UK EMRSA-15	13 (93)	1 (7)	14	No	A	1997
E19	13 (100)	0 (0)	13	Yes	C	1997
Pori clone (O15)	8 (62)	5 (38)	13	No	A	1993
E20	12 (100)	0 (0)	12	Yes	C	1998
Iberian clone (E6, E7, E10, O8)	11 (92)	1 (8)	12	Yes	B	1991
O25	12 (100)	0 (0)	12	Yes	B	1997

^a^HVR, hypervariable region; E, epidemic, strain isolated in more than one health-care facility; O, outbreak, strain isolated in one health-care institute. ^b^Resistance to more than three antibiotic groups in addition to β-lactams.

**Figure:**
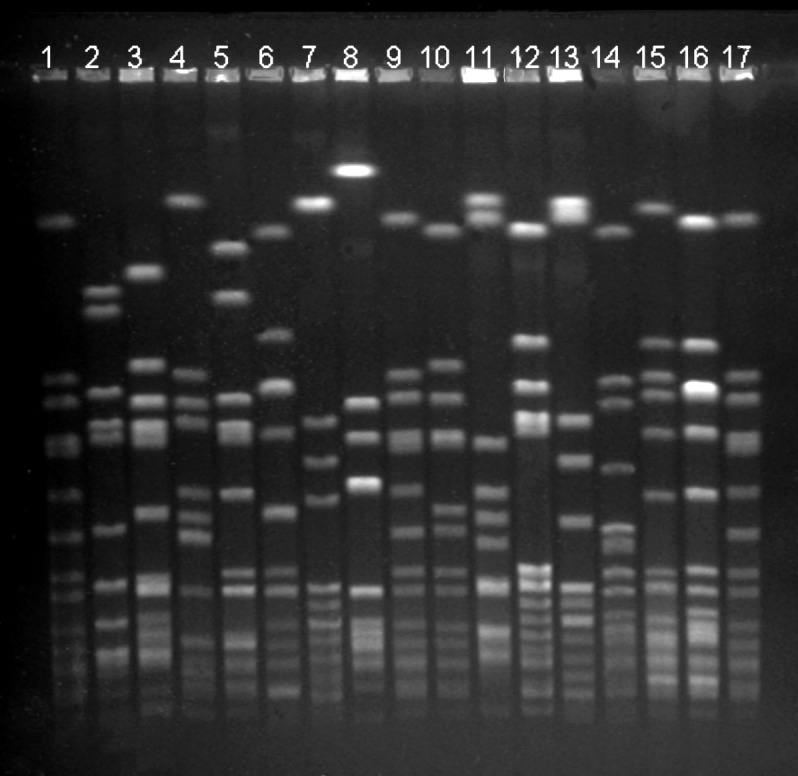
Pulsed-field gel electrophoresis (PFGE) profiles of the 14 most common methicillin-resistant *Staphylococcus aureus* (MRSA) strain types identified, Finland, 1997–1999. Lanes 1, 9, 17: *S. aureus* NCTC 8325 (molecular weight marker); lanes 2–4: strain types associated with community acquisition (Mikkeli clone, E22, E31); lane 5: E1; lane 6: E24; lane 7: E5; lane 8: Kemi clone; lane 10: E27; lane 11: UK EMRSA-15; lane 12: E19; lane 13: Pori clone; lane 14: E20; lane 15: Iberian clone; and lane 16: O25.

Of all strains isolated from persons who had no hospital contact, 94% were nonmultiresistant. In addition, of the 14 most common strain types, all 7 nonmultiresistant strains, but none of the multiresistant strains, showed HVR type A (Table 2). Of the 56 sporadic MRSA strains, 41% were nonmultiresistant, including all but one of the strains isolated from persons without connections to hospitals (Table 1).

## Discussion

Our population-based study showed that from 1997 to 1999 one fifth of all Finnish MRSA isolates came from persons who had no connection to health-care facilities, suggesting that these MRSA isolates may be community acquired. Three strain types identified by phage typing, PFGE, and ribotyping were associated with community acquisition, and none of these strain types were multiresistant.

To our knowledge, this is the first report of community-acquired MRSA on the national level. Previous reports have focused on single health-care institutions or certain restricted areas (*1*,*3*,*6*,*12*,*18*,*19*). Both data sources used in our study, the surveillance and typing scheme of MRSA and the hospital discharge register, were nationwide (*20*). The availability of national identity codes allowed us to link the two data sources and to study one MRSA isolate per person. The number of isolates routinely typed and verified as MRSA was equal to that of MRSA isolations reported to the National Infectious Disease Register, suggesting that the isolates of MRSA available for typing were nationally representative.

Community-acquired MRSA can be classified into the following categories: discharged hospital patients with MRSA, nursing-home residents with MRSA, MRSA transmitted to nonhospitalized patients, and MRSA arising de novo in the community (*7*). The first three categories include MRSA isolates of health-care facility origin, which are thought to represent a limited number of different genotypes, disseminate clonally, and express resistance to multiple antibiotics (*1*,*21*–*23*). De novo MRSA strains, in contrast, are thought to arise through acquisition of *mec* DNA into a previously susceptible *S. aureus* genotype (*7*,*9*,*24*). Our study focused on the last two categories. We first identified nonhospitalized MRSA-positive persons and thereafter compared their strains with those found in hospitalized patients.

Based on our study, the proportion (21%) of community-acquired MRSA was relatively high in Finland. However, the definition of community-acquired MRSA is not straightforward. The definition classically includes MRSA isolated outside hospital settings or from a patient within 48 to 72 hours of hospital admission. Because of the long-term persistence of MRSA colonization (*25*), contacts with hospitals or nursing homes before MRSA isolation should also be taken into account (*6*,*19*,*26*). Therefore, our definition of community acquisition covered a 2-year time period without a health-care facility contact before the MRSA isolation. If the cut-off period had been 1 year, the proportion of community-acquired isolates would have been 26%. If the questionnaire survey on the additional background data had not been performed, the proportion would have been 29%. Although this questionnaire survey was not comprehensive, it allowed us to characterize the persons who had no reports in the discharge register and to identify persons with a foreign or ongoing hospital contact.

Most community-acquired MRSA strains were nonmultiresistant, and children were more likely to have a community- than a hospital-acquired MRSA. These findings agree with those of previous reports, suggesting that nonmultiresistant MRSA is emerging as an important pathogen in the community (*1*,*18*,*22*,*23*). The majority (64%) of community-acquired strains were isolated on a clinical basis. However, one third of all community-acquired strains were isolated because of screening of persons exposed to a known MRSA carrier, most of whom were family members. Most exposed persons had the same strain type as their contacts (data not shown).

Among the 14 most common strain types, which represent 80% of all MRSA isolates, we identified 7 nonmultiresistant strain types showing a hypervariable region hybridization pattern A. Three of these strain types were associated with community acquisition and represented more than half of all community-acquired strains. In addition, three other strain types were frequently found in persons without connections to hospitals. The only exception was the internationally recognized UK EMRSA-15 (*27*), which was isolated mainly from patients or health-care workers who had recently returned from hospitals abroad. In contrast, the multiresistant strain types, including the UK EMRSA-16 (*28*) and the Iberian clone (*29*), were almost exclusively found in persons who had contacts with hospitals.

Despite the fact that the HVR type A seems to coincide with nonmultiresistance, the hypervariable region itself has not been shown to contain any antibiotic resistance markers (*17*,*30*,*31*). Some of the multiresistant strains actually have deletions in this area (*31*). The hypervariable region has been analyzed by polymerase chain reaction and sequencing to distinguish different *mec* DNA types (*32*,*33*). The method identified five different subclones in 50 isolates representing one epidemic strain in Germany (*33*).

Our study had several limitations. First, some of the MRSA isolates classified as community acquired may have been isolated from nursing-home residents, since not all Finnish nursing homes report to the National Discharge Register. The possibility of misclassification concerns a small number of isolates, since only 10 of all persons with community-acquired MRSA were >64 years of age. Second, the differences in local sampling policies may affect the number and type of community-acquired MRSA identified. National guidelines for MRSA prevention in Finland are primarily directed to hospital use, and sampling and screening policies in community setting are not specified. Third, we did not gather clinical data and risk factors (*6*,*34*,*35*) other than previous hospital stays for MRSA acquisition. Further information should be collected from persons with community-acquired MRSA to develop a hypothesis on risk factors specific for community acquisition.

In conclusion, a large proportion of MRSA-positive persons may have acquired their strains outside the hospital setting, and their MRSA strains were nonmultiresistant, showed an HVR type A, and differed genotypically from epidemic strains found in hospitalized patients. None of the epidemic multiresistant hospital strains were prevalent in nonhospitalized persons. Our findings suggest that MRSA may also emerge as a community-acquired pathogen as a consequence of horizontal acquisition of the *mecA* gene to a previously susceptible *S. aureus* strain type.
